# Herbicidal weed management practices: History and future prospects of nanotechnology in an eco-friendly crop production system

**DOI:** 10.1016/j.heliyon.2024.e26527

**Published:** 2024-02-22

**Authors:** Santosh Kumar Paul, Santa Mazumder, Ravi Naidu

**Affiliations:** a Global Centre for Environmental Remediation (GCER), ATC Building, The University of Newcastle, Callaghan, NSW 2308, Australia; b CRC for Contamination Assessment and Remediation of the Environment (crcCARE), ATC Building, The University of Newcastle, Callaghan, NSW 2308, Australia; c Agronomy Division, Bangladesh Agricultural Research Institute (BARI), Joydebpur, Gazipur 1701, Bangladesh; d Sher-E-Bangla Agricultural University, Dhaka-1207, Bangladesh

**Keywords:** Herbicide, Weeds, Nanotechnology, Controlled release formulation

## Abstract

Weed management is an important aspect of crop production, as weeds cause significant losses in terms of yield and quality. Various approaches to weed management are commonly practiced by crop growers. Due to limitations in other control methods, farmers often choose herbicides as a cost-effective, rapid and highly efficient weed control strategy. Although herbicides are highly effective on most weeds, they are not a complete solution for weed management because of the genetic diversity and evolving flexibility of weed communities. The excessive and indiscriminate use of herbicides and their dominance in weed control have triggered the rapid generation of herbicide-resistant weed species. Moreover, environmental losses of active ingredients in the herbicides cause serious damage to the environment and pose a serious threat to living organisms.

Scientific advances have enabled nanotechnology to emerge as an innovation with real potential in modern agriculture, adding a new dimension in the preparation of controlled release formulations (CRF) of herbicides. Here the required amount of active ingredients is released over longer periods of time to obtain the desired biological efficacy whilst reducing the harmful effects of these chemicals. Various organic and inorganic carrier materials have been utilised in CRF and researchers have a wide range of options for the synthesis of eco-friendly carrier materials, especially those with less or no toxicity to living organisms. This manuscript addresses the history, progress, and consequences of herbicide application, and discusses potential ways to reduce eco-toxicity due to herbicide application, along with directions for future research areas using the benefits of nanotechnology.

## Highlights

• Weed management is always an important aspect of crop production to ensure quality and higher production.
• Chemical weed management stand first due to their cost-effectiveness, rapid action and easy application methods.
• The burst release of active ingredients from available formulations causes eco-toxicity through leaching and runoff.
• Controlled release formulations using nanotechnology can compensate the problems of current formulations.
• Research must be focus on synthesis of eco-friendly carrier materials for controlled release formulations.

## Graphical abstract

**Figure:**
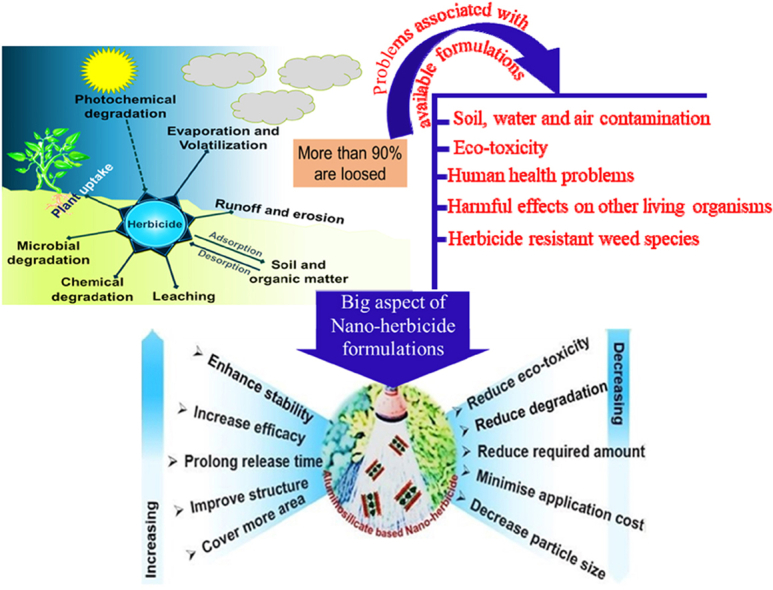

## 1 Introduction

Weeds are considered as the most harmful pests (compared to birds, animals, insects, and pathogens) in crop production, and cause significant losses in terms of yield and quality. Therefore, weed management is an important aspect of crop production because weeds have such a dynamic and stubborn nature ([1], Santosh Kumar [2,3,4]). Generally, weeds compete with crops for light, water, nutrients, and space because they exist at the same trophic level of the food chain as crop plants [1,[5], [6], [7]]. As a result, a significant yield loss occurs. This has implications for food security as it puts pressure on the food supply for the world's population. Therefore, weed management is an essential process in agricultural systems. In weed science, “weed management” is a more appropriate term than “weed control”. Weed management involves keeping the weed population below the weed threshold level [8,9].

**Fig. 1:**
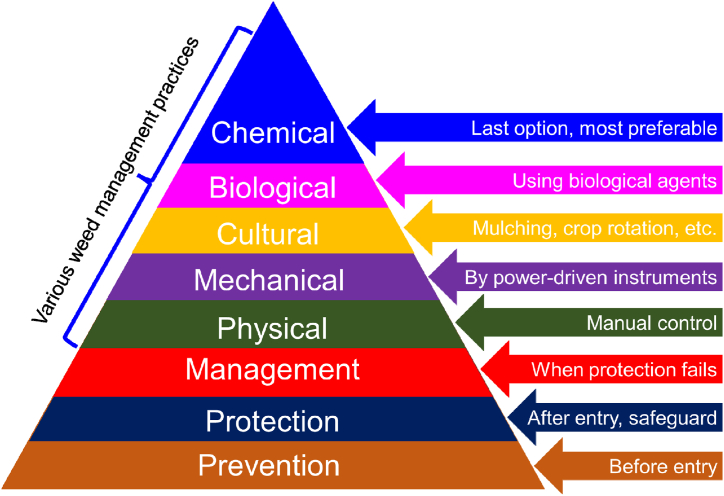
Pyramid of effective weed control strategies.

For sustainable weed management practices, it is important to know and identify the weed threshold level of each specific crop [1,[10], [11], [12]]. So far, various approaches are being used to control weeds (Fig. 1), including preventive, physical, cultural, mechanical, biological, and chemical methods [8,[12], [13], [14], [15]]. Of all possible weed management practices, preventive control is the key strategy and the first consideration for new weed communities, especially invasive species [8]. However, it is quite difficult to prevent the dispersion of weed seeds due to weak quarantine systems globally. Apart from certain countries, such as Australia, there are no restrictions on taking seeds from one place to another. Moreover, animals, birds, and the wind also disperse weed seeds over long distances.

The physical methods have limitations, including labour scarcity and high labour costs. Mechanical weed management is not always feasible due to the additional costs required for fuel and equipment. Moreover, these practices greatly depend on favourable weather conditions. In addition, some perennial weeds can regenerate from broken parts of roots and shoots, making it difficult to control weeds completely [16]. As a result, farmers are shifting to chemical weed management practices, and they often prefer to apply herbicides due to their cost-effectiveness, rapid action, high weed control efficiency, and easy application. Farmers tend not to adopt non-chemical weed management practices if they still have the option to use herbicides. Of the various approaches for weed management, the introduction of herbicides has added a new dimension and revolutionized modern agriculture [17,18,19].

**Fig. 2:**
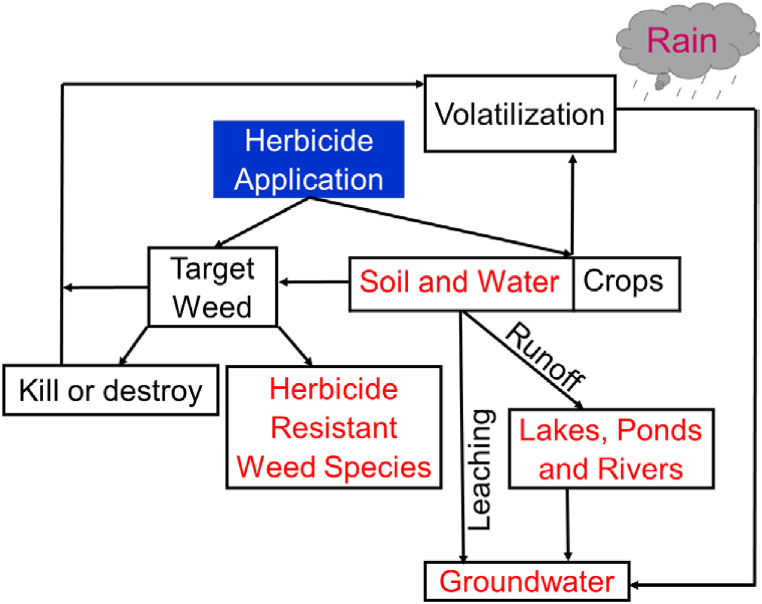
Effect and consequences of herbicides.

So far, herbicides have been the dominant tool for weed control due to their selective weed control properties as well as their ability to control weeds in a short time, even over large areas, in a cost-effective manner [12,18]. Although herbicides are highly effective on most weeds, they are not a complete solution for weed management due to the genetic diversity and evolving flexibility of weed communities. Hence, the evolution of herbicide-resistant (HR) weeds has become a major concern in recent times (Fig. 2). Furthermore, the excessive and indiscriminate use of herbicides and their dominance in weed control has triggered the rapid generation of HR weeds [9,17,20]. Moreover, the environmental losses of the active ingredients of herbicides can cause serious damage to our environment and threaten all living organisms [11,[21], [22], [23], [24],^19^].

**Fig. 3:**
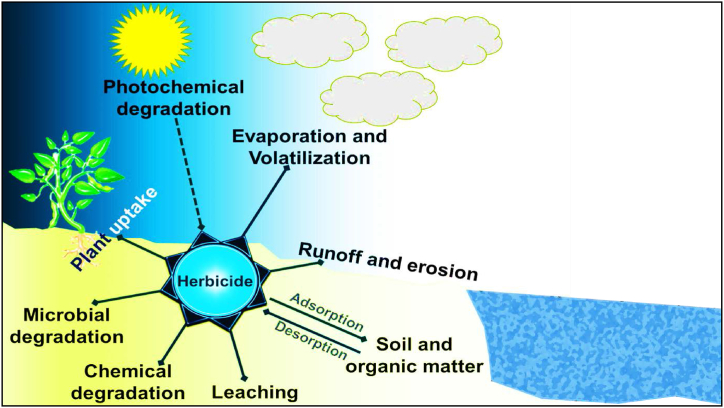
Environmental fates of herbicides.

The application of herbicides has become a global issue because of the problems they cause for the environment and the food chain [3,5,8,25]. It has been reported that >90% of applied pesticides (including herbicides, insecticides, fungicides) do not reach the target pest and are lost in different ways, resulting in water, soil, air, and food contamination [[26], [27], [28]]. The ways herbicides are lost are illustrated below in Fig. 3.

To compensate for the negative impacts of herbicides, integrated weed management practices that include all possible methods of control are encouraged. The main goal of integrated weed management is to reduce reliance on herbicides, but farmers only adopt non-chemical strategies for weed control when there is no other option ([11,18].). In this context, the development of new herbicide formulations is highly desirable and has long been an active field of research so that the problems associated with commercial herbicides can be resolved. This manuscript focused on the history, progress, and consequences of herbicide application, and discussed potential ways to reduce eco-toxicity due to herbicide application. Selection of suitable carrier materials stands first to synthesis effective CRF of herbicide. This review directed the ways to use eco-friendly carrier materials for CRF to reduce eco-toxicity due to chemical herbicides. Future research directions are made on synthesising eco-friendly carrier materials for CRF and investigation of long-term toxicological effects of synthesised new formulations on soil flora and fauna.

## 2 Herbicides

### 2.1 An overview

Herbicides are a subcategory of pesticides used to kill weeds. The word “herbicide” is derived from two Latin words, firstly, “herba”, meaning “plant”, and secondly, “caedere”, meaning “to kill”. Thus, herbicides can be defined as chemicals that kill plants [29]. The Weed Science Society of America (WSSA) has accepted the definition of herbicide as “a chemical substance or cultured organisms used to kill or suppress the growth of plants” [30]. Simply put, herbicides can be defined as any chemical substance that is toxic to plants and used to control, destroy, or inhibit undesirable vegetation, especially weeds in crop fields. Herbicides are classified/grouped in various ways according to factors such as the timing of application, their selectivity, the site of application, their mode of action, and so on (Table 1). Generally, pesticide formulations (including herbicides, insecticides, fungicides, etc.) are prepared with two distinct materials: (i) active ingredients, which are the main substances used to repel, mitigate, prevent, or destroy the target pest; and (ii) inert ingredients (materials or carriers), used as stabilisers and to improve the performance and usability of the pesticide. The compatibility of the active ingredients and inert materials is very important for synthesising an effective and desirable pesticide formulation. If the active ingredients are strongly bonded to the carriers and cannot be released spontaneously after application, the formulation may be less effective and not suitable for commercial application. Conversely, if the active ingredients are loosely bonded with the carrier and are rapidly released after application, the formulation will not be commercially acceptable for toxicological reasons. Therefore, the release of the active ingredients is an important factor in pesticide formulation, and it has become a widely accepted question for commercial pesticide development.

**Table 1:** Classification of herbicides.

Sl. No.	Types	Properties	Example
A	***Based on selectivity***		
1	Non-selective herbicides	Non-selective/broad-spectrum herbicides kill all or most plant species	Diuron
2	Selective herbicides	These herbicides kill specific weed species, leaving the desirable vegetation relatively unharmed	2, 4-D, Glyphosate
B	***Based on mode of action***		
1	Contact herbicide	Contact herbicides can kill only the chemically contacted plant parts	Paraquat
2	Systemic/translocated herbicide	Systemic herbicides are absorbed by plant foliage or roots and translocated (moved) throughout the plant	Glufosinate ammonium, 2,4-D, Glyphosate
C	***Based on site of application***		
1	Soil-applied herbicides	Herbicides are applied to the soil	Sulfonylureas, Triazines
2	Foliage-applied herbicides	Herbicides are applied to the foliage	Most of the phenoxy acids
D	***Based on time of application***		
1	Pre-sowing or pre-planting herbicides	Some herbicides need to be applied to the soil immediately before planting, while others need several weeks prior to planting	Fluchloralin, Alachlor
2	Pre-emergence herbicides	Herbicides are normally applied after sowing but before the emergence of crops, weeds or both	Simazine, Atrazin, Butachlor, Pendimethalin
3	Post-emergence herbicides	Herbicides are applied after the emergence of weeds, crops or both	2, 4-D, Diquat, Paraquat, Isoproturon
E	***Based on ionic properties***		
1	Anionic herbicides	Negatively charged and proton acceptor	2,4-D, Metolachlor
2	Cationic herbicides	Positively charged and proton donor	Diquat, Paraquat

### 2.2 Progress and development of herbicide formulations

The use of herbicide in weed management is not a recent trend and in fact it has a long history. Application began in the early 1900s, with inorganic copper salt the first chemical to be used as a herbicide [13,31]. Then in 1906, carbon bisulfide was introduced as a soil fumigant to control field bindweed and Canada thistle, followed by petroleum oil (kerosene) being introduced as an organic weed killer along irrigation ditches in 1914 [16]. Though kerosene has soil-sterilising effects and arsenic is considered more poisonous, both substances were nevertheless used in weed management later. Arsenic trichloride was marketed as “KMG” to kill the weed known as “morning glory” in the 1920s. Certain other chemical compounds, such as sodium chlorate, sulfuric acid, etc., were also becoming popular in the early 1900s [8,13,16,31]. In 1923, sodium chlorate was introduced in France for the control of field bindweed, despite its high fire risk. On October 4, 1929 the National Research Council of Canada organised a conference and the chairman concluded that spraying of sulfuric acid could be considered for weed management practice [8]. During the 1930s, sulfuric acid was introduced in Britain for successful weed control strategies. It was, and continues to this day, to be an effective herbicide, but it is very corrosive and harmful to people as well as dangerous to work with. Common salt was also used in weed control, however, it has an adverse effect on soil properties and diminishes the productivity of soil [16].

The introduction of 2,4-dichlorophenoxy acetic acid (2, 4-D) in the 1940s marked an important new dimension in weed management. A synthetic plant hormone known as MCPA (2-methyl-4-chlorophenoxyacetic acid) was introduced in 1942 during World War II, and opened a new era that demonstrated site-specific control of weeds [31]. It is selective in nature and employed to control broadleaf weeds. This systemic herbicide acts similarly to the plant growth hormone auxin. With continuous research being undertaken, several other new active ingredients including diquat, paraquat, diuron, atrazine, fenac, trifluralin, and ioxynil were introduced during the 1950s and 1960s [32]. After considering their cost-effectiveness ratios, the innovation and application of herbicides, the WSSA approved 25 herbicide active ingredients from 1940 to 1950, and this number reached 100 in 1969 [33]. It has been reported that in 2002 there were 204 different herbicides in use [34]. More recently, the WSSA approved 315 common herbicides that are available on the market [25,35], which possess diverse modes of action (Table 2).

**Table 2:** Groups of herbicide active ingredients and their mode of action.

Group Name	Mode of Action
Group A	Inhibitors of fat synthesis/AACase inhibitors
Group B	ALS inhibitors
Group C	PS ll inhibitors
Group D	Inhibitors of microtubule assembly
Group F	Bleachers: PDS inhibitors
Group G	PPO inhibitors
Group H	Bleachers: HPPD inhibitors
Group I	Disrupters of plant cell growth
Group J	Lipid synthesis inhibitors (not ACCase inhibitors)
Group K	VLCFA inhibitors
Group L	PS l inhibitors
Group M	Inhibitors of EPSP synthase
Group N	Inhibitors of glutamine synthase
Group O	Inhibitors of cell wall synthesis
Group Q	Bleachers: inhibitors of carotenoid biosynthesis unknown target
Group R	DHP inhibitors
Group Z	Herbicides with unknown and probably diverse sites of action

**Fig. 4:**
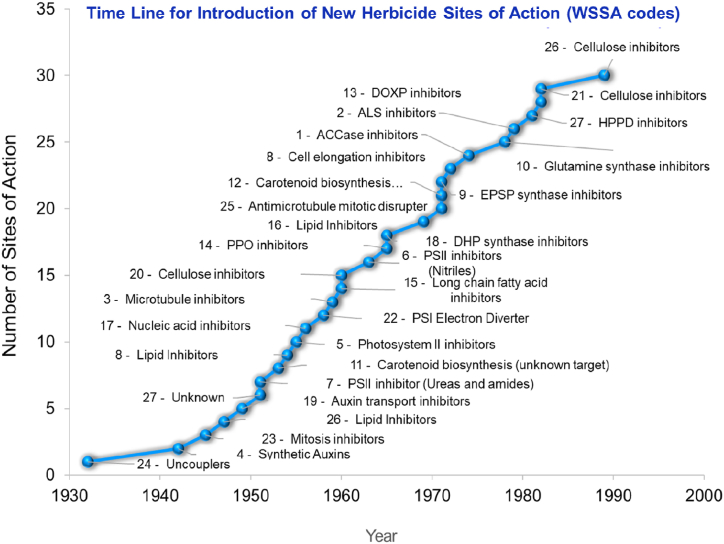
Introduction of various herbicides with diverse sites of action. Source: [36].

A number of herbicides with diverse sites of action have been introduced over the last few decades (Fig. 4). It is notable that no new herbicide active ingredients have been introduced over the last 30 years. Thus, considerable time is required to improve current herbicide formulations to reduce their negative effects.

Herbicides are categorised into different chemical families according to the chemical compositions of their active ingredients and their modes of action. Various herbicide companies are marketing products using different brand names, but essentially with the same/similar mode of action and active ingredients.

### 2.3 Global trend of herbicide application

**Fig. 5:**
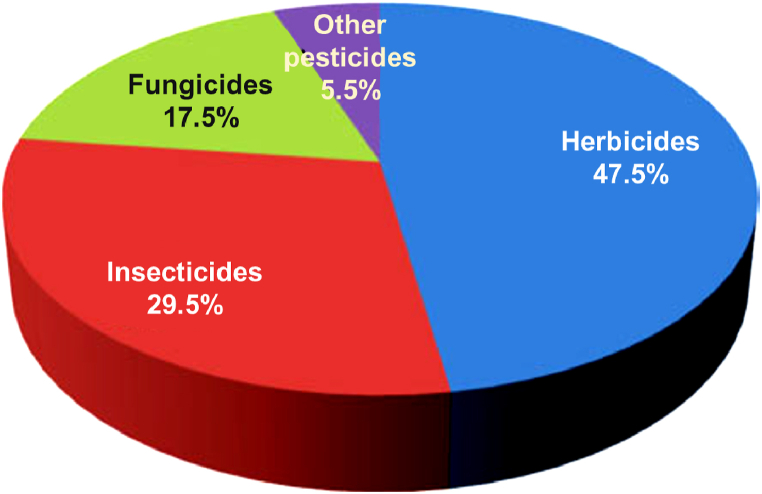
Worldwide percentages of different pesticides. (adapted from [39].

The use of pesticides (herbicides, insecticides, fungicides, and others) to control agricultural pests has dramatically increased since the Green Revolution of the early 1960s [31,37]. Worldwide annual consumption of pesticides has risen dramatically since then, and a few years ago reached nearly four million tonnes [37,38] with herbicide being the most widely applied pesticide (Fig. 5).

It is reported that 45% of pesticides are used in Europe alone, followed by 25% in the United States, while 25% is consumed by rest of the world, with India using just 3.75%. The dose of pesticide application varies significantly in different countries, 12.0 and 6.6 kg/ha in Japan and South Korea, respectively, whereas India is only 0.5 kg/ha [40]. Some reports state that globally there has been a decline in the rate of use of other pesticides, though not herbicides [38]. In 1990, the worldwide total applied active ingredients of insecticides reached 0.3 million tonnes, but in 2014 it was about 0.25 million tonnes. Conversely, globally the total used herbicidal active ingredients were 0.5 million tonnes in 1990, with an increase of about 0.8 million tonnes in 2014 [38]. Based on this, it can be concluded that herbicide usage is increasing rapidly due to a lack of adequate non-chemical-based weed control methods [41].

In 1997, the United States used 1 billion pounds (0.45 billion kgs) of various pesticides, of which 461.4 million pounds (207.63 million kgs) (>47%) were herbicides [16,42]. The costs associated with pesticide application are gradually increasing throughout the world, and herbicides rank in first place [16,42,43]. Annual expenditure on herbicide application between 1960 and 1999 was the highest compared to all other pesticides in the United States [44].

### 2.4 The consequences of herbicide application

**Fig. 6:**
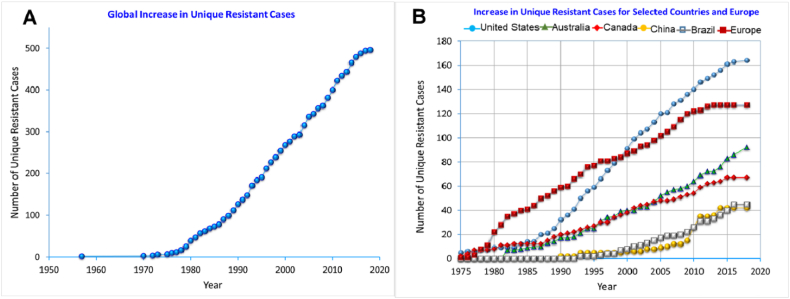
Number of herbicide-resistant weed species (A) global situation over time and (B) selected countries where rapid increase of HR species is evident. (adapted from [36].

In crop production, while herbicides play an important role in controlling weeds, they do serious harm to the environment, humans, flora, and fauna. Highly soluble herbicides are easily available after application and move through runoff and leaching, contaminating the soil as well as surface and groundwater [22,[45], [46], [47]]. The presence of persistent pollutants in drinking water increases risks to human health [21,37]. Moreover, some herbicides have a long-term residual effect, and their absorption by plants is considerable. Accumulation of these toxic residues in agricultural products accelerates their mixing in our food chain, which endangers the health of humans and animals [22]. In crop cultivation, the major concern regarding herbicide application is weed resistance to a particular herbicide. Continuous application of herbicides with similar modes of action over a longer period of time, as well the same mode of action in the same field, leads to more HR weed species [17,23,24]. Currently, weed resistance to herbicides is a major concern because this is increasing all over the world. In 1995, the 6th International Survey of Herbicide-Resistant Weeds documented 183 weed biotypes resistant to herbicide (124 species) in 42 countries [17]. This number is now approximately 500 weed biotypes resistant to herbicides worldwide (Fig. 6A). However, the developed countries are more prone to HR in most weeds. The highest levels of HR have occurred in the United States, followed by Australia, China, and parts of Europe. In 2012, there were 30 HR weed species in Australia [48], although today the number is more than 90 (Fig. 6B). Over time, this is increasing in the developing countries.

**Fig. 7:**
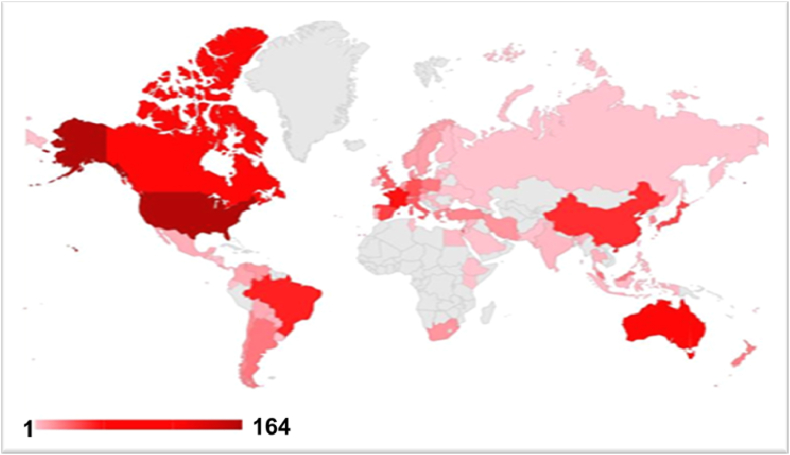
World scenario of weed resistance to herbicides (adapted from [36].

The generation of HR weed species is increasing concomitantly with the high dosages of herbicide application throughout the world. Herbicide application has risen tremendously compared with previous decades, especially in developed countries, producing a large number of HR weed species (Fig. 7).

**Fig. 8:**
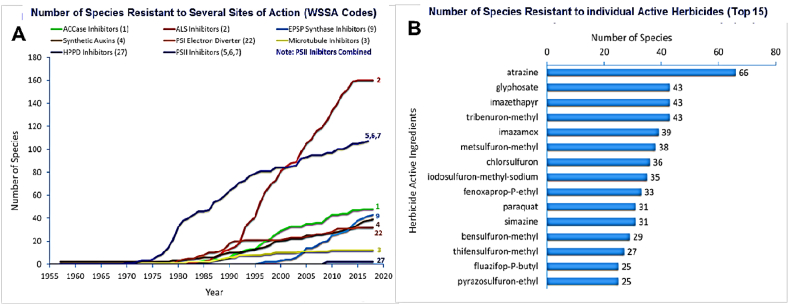
Number of weed species resistant to (A) various sites of action and (B) different active ingredients (commonly used top 15) (adapted from [36].

A number of herbicides with different sites of action, including ACCase inhibitors, photosynthesis inhibitors, ALS inhibitors, PSII inhibitors, and HPPD inhibitors, are commonly used in crop fields. However, it is alarming that many weed species are now already very resistant to some of these, and the number is increasing with time (Fig. 8A). The numbers of weed species resistant to different herbicide active ingredients (top 15 herbicides) is depicted in Fig. 8B.

**Fig. 9:**
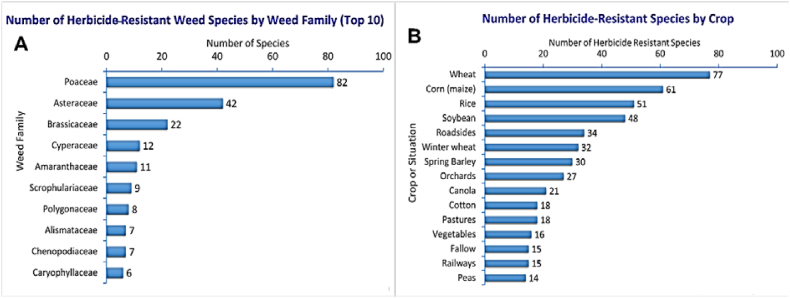
A number of weed species are resistant (A) from several botanical families and (B) some crop-associated weeds (major and commonly cultivated agricultural crop-associated weeds) (adapted from [36].

The number of HR weed species from various botanical families is increasing as time passes, with the weed species of the Poaceae family increasing faster than other botanical families (Fig. 9A). Most weeds found around the world are crop-specific and grow in association with a specific crop. The weed varieties grown in association with major and commonly cultivated agricultural crops are able to resist available commercial herbicide active ingredients. The increasing numbers of HR associated weeds are shown in Fig. 9B.

### 2.5 Possible ways to reduce herbicidal eco-toxicity

With reference to weed management practices, herbicides have for decades represented the most common and reliable method for crop growers throughout the world. Their cost-effectiveness and ease of application as well as their rapid and high weed control efficiency, mean that many farmers believe their benefits outweigh the problems associated with their use [17,18]. For this reason, it is difficult to persuade famers to shift from herbicides to other methods of weed control. Integrated weed management practices could be an alternative way to reduce the reliance on herbicides [8,18]. Some non-chemical-based methods including mowing, prescribed burning, or timely grazing, which can control weed populations to a certain extent [49]. Use of genetically modified crops does play an effective role in non-chemical weed management practices [20]. However, these techniques are all time-dependent and not feasible everywhere. A few years ago, Moss [18] articulated 16 reasons for farmers’ reluctance to use non-chemical weed control methods; consequently, it is crucial to improve current conventional herbicide formulations, and urgently so.

Nanotechnology has emerged as a potential innovation in modern agriculture and can be applied to various preparation strategies of nano-herbicide formulation, adding a new dimension to controlled release formulations (CRF). A nano-herbicide is a subcategory of nano-pesticides with the ability to kill weeds. Being in the nano-size range, the surface area of a nano-herbicide is comparatively high, so less is required to cover a greater area, which ultimately reduces reliance on chemicals. Moreover, this supports better absorption of active ingredients by plants [50]. The long-term release behaviour of the nano-herbicides may improve their efficacy in controlling the target weed, and they cannot wash into runoff or be leached out immediately after application, preventing environmental toxicity.

The prefix “nano” means one-billionth of a metre, which is approximately 1/80,000 of a human hair diameter or ten hydrogen atoms wide [51]. Nano-herbicides are a specific formulation of herbicides where the active ingredients are either delivered through nano-size (1–100 nm) carrier materials or oriented within a nano-structure [52,53]. Nano-herbicide formulations have so far exhibited the potential to manage agricultural weeds effectively compared to conventional formulations [24]. Subsequently, nano-delivery systems have made it possible to formulate more dynamic controlled release herbicide formulations so that the active ingredients can be delivered to the target in a “smart” way [54] with improved features [55,56].

The aim of nano-formulation is similar to other herbicide formulations, although better efficacy than existing commercial formulations is expected. The main distinguishable properties of nano-formulations are their size specificity, where the upper limit is not more than 100 nm [57]. Nano-formulations are categorised into different groups such as nanoparticle/nanocide, nano-delivered, nano-composited and nanodroplet-oriented substances [58]. Of these categories, nano-delivered formulations are preferable as they can be prepared by loading active ingredients onto nano-sized carrier materials through different ways such as adsorption, attachment, entrapment, and encapsulation, which protects the active ingredients from premature degradation and allows the slow release of the active ingredient [59].

CRF are considered an effective pest management strategy, where the required amounts of active ingredients are released over a longer period of time to obtain the desired biological efficacy, and reduce the harm these chemicals do to the environment [60]. CRF were initially used in a drug delivery system in 1952 [61], and some synonymous terms were introduced to describe CRF, including “extended release”, “sustained release”, “timed release”, etc. [62]. In drug delivery systems, capsules or pills were commonly used before the 1950s, hence the introduction of CRF has significantly changed medical practices due to their advanced features compared to existing formulations. However, the use of CRF in herbicide delivery systems began at a later stage with microemulsion or microencapsulation processes. The particle sizes of these formulations were about 250 times smaller compared to typical herbicide particles [57,63]. Currently, several pesticide companies are marketing microemulsion formulations using a variety of names. For instance, Syngenta is marketing Primo MAXX which has been used by golf course superintendents since 1993 [57,64]. Various commercially available herbicide formulations are listed in Table 3.

**Table 3:** Different types of commercially available herbicide formulations.

Formulation Types	Example(s)
Soluble powder (SP)	2, 4-D sodium salt, Roundup Pro Dry, Dalapon
Soluble concentrates (SC)	2, 4-D amine aster, Diquat, Paraquat
Wettable powder (WP)	Atrazine 80%, Simazine 50%, Isoproturon 70WP
Flowable (F or L)	Atrazine 4 L, Princep 4 L
Liquid suspension (LS)	Atrazine, Cyprazin, Nitralin
Solution (S)/concentrated solution (C or LC)	Banvel/Clarity 4 S, Roundup Pro Concentrate
Emulsifiable concentration	2, 4-D ester, Alachlor, Nitrofen
Granules	Granules of Butachlor, 2, 4-DEE
Microcapsule	Primo MAXX

When considering eco-friendly crop production systems, agriculture researchers now show more interest in CRF that are herbicide-based. Nano-herbicide formulations using non-toxic, eco-friendly carrier materials are a good and safe method for effective weed management [[65], [66], [67]]. In this context, various organic and inorganic carrier materials, including clay minerals, layered double hydroxides, polymers, lipids, inorganic porous materials, metals, and metal oxides have been utilised for the preparation of CRF [27,59,68]. The use of different carrier materials in CRF of various agrochemicals and their improved features are presented in Table 4. However, the selection of carrier materials is very important to make the delivery system sustainable. Selection primarily depends on the type of desired formulation, the physicochemical properties of the target herbicide as well as its compatibility with the carrier materials [59]. For example, non-biodegradable polymers are not suited for environmental application, whereas biodegradable polymers are not stable for long periods of time. In this case, inorganic carriers are better. Nonetheless, these carriers have certain controversial issues to overcome with respect to their fate in the environment, in that metals and their oxides may have a toxic effect on the environment and living creatures.

**Table 4:** Application of various carrier materials in CRF/SRF of pesticide(s) with improved features.

Sl no.	Carrier material	Pesticide(s)	Formulation Type	Improved Features due to Nanoencapsulation	Reference
***Polymer-based:***					
1	Alginate	Atrazine and Diuron	Nanocomposite	Helps in prolonged release of atrazine and diuron	[69]
2	Alginate	Clomazone	Hydrogel	Clomazone release from dry capsules was slower than the alginate-hydrogel formulation	[70]
3	Alginate-chitosan	Paraquat	Nanoparticles	Alters the release profile of paraquat and reduces negative effects on soil	[71]
4	Ethylcellulose	Norflurazon	Microspheres	Microspheres were able to provide a gradual and sustained release of norflurazon	[72]
5	Ethylcellulose	Norflurazon	Microspheres	Reduces the release rate and persists for longer in the soil (t1/2 values were 7.72 and 30.83 weeks for CF and MEFs)	[73]
6	Ethylcellulose	Chlorsulfuron	Coated granules	Ethylcellulose-coated CRF reduce the release rate of chlorsulfuron	[74]
7	Chitosan	Dichlorprop	Nanoparticles	The behaviour of enantioselective compounds may change when interacting with other chiral receptors	[75]
8	Chitosan	Dichlorprop	Nanoparticles	In the absence of chitosan, the (R) enantiomer was more toxic than (S)-enantiomer to *Chlorella pyrenoidos*. Conversely, the (R)-enantiomer was less toxic in the presence of chitosan	[76]
9	β-Cyclodextrin	4-chloro-2-methylphenoxy acetic acid (MCPA)	Microencapsulation	Functionalised β-CDs showed an increase in the binding affinity of MCPA in relation to β-CD	[77]
10	β-Cyclodextrin	2,4-dichlorophenoxyacetate (2,4-D)	Microencapsulation	Removal of 2,4-D was improved in the presence of β-CD	[78]
11	β-Cyclodextrin	Chlorpropham	Microencapsulation	Complexed with β-cyclodextrin, the thermal stability and water solubility of chlorpropham significantly improved	[79]
12	Cellulose	Alachlor and Metolachlor	Microcapsules	Formulation of alachlor with 9-month-old ethyl cellulose was more active than the commercial formulation and exhibited controlled release properties	[80]
13	Alginate/chitosan	Paraquat	Microcapsules	The adsorption capacity of alginate/chitosan bilayer was high and could be especially useful for simultaneous adsorption of different substances using the same material	[81]
14	Chitosan	Atrazine	Nanocapsules	The release kinetics profile of the herbicide was altered when the nanocapsules were coated	[82]
15	Chitosan	Paraquat	Nanoparticles	Humic substances can decrease the toxic effects of chitosan nanoparticles containing paraquat	[83]
16	Poly(ε-caprolactone)	Atrazine	Nanogranules		[84]
17	Polylactic acid (PLA)	Lambda-cyhalothrin	Microcapsules	A good UV and thermal stable formulation with similar efficacy as a commercial formulation	[85]
18	Poly(ε-caprolactone)	Atrazine	Nanogranules	The encapsulation efficiency was 65% and stable compound evident when coated with chitosan; has potential weed control efficiency	[86]
***Lipid-based:***					
19	Beeswax (chitosan-coated solid lipid)	Deltamethrin	Nanoparticles	Reduces photodegradation with higher efficacy than commercial formulation	[87]
20	Corn oil (liquid oil)	Deltamethrin	Nanocarriers	Higher payload with slower release rate and protection from photodegradation	[88]
21	Corn oil (chitosan-coated)	Deltamethrin	Nanoemulsions	Higher loading capacity with slower release rate and higher photodegradation capacity	[89]
22	Compritol 888 (lipid)	Gamma-cyhalothrin	Nanoparticles	Has same insecticidal activity and reduces toxicity	[90]
23	Compritol 888 (lipid)	Oil of *Artemisia arborescens*	Nanoparticles	Higher physical stability and reduced rapid evaporation	[91]
***Metal and metal oxide-based:***					
24	Silica	2,4-D and picloram	Nanogel	Supports slow-release of herbicides 2,4-D and picloram in a controlled way	[92]
25	Silica	Fipronil	Nanocapsules	High encapsulation efficiency with sustained release performance	[93]
26	Silica	Metalaxyl	Nanospheres	Excellent carriers for CRF and protects the loaded molecules from enzymatic degradation	[94]
27	Silica	Imidacloprid	Nanoparticles	Good adsorption capacity and slow-release and protection against premature degradation	[95]
28	Silica	Tebuconazole	Nanospheres	High loading efficiency (45%) and potential interaction between silica and pesticide with sustained release capacity	[96]
29	Silica	Uniconazole	Nanoparticles	Reduced release rate and showed growth retardation effect	[97]
30	Silica	Silica nanoparticles	Nanoparticles	Novel insecticide which creates a barrier in water circulation in an insect's body	[98]
31	TiO_2_	TiO_2_/Zn	Nanoparticles	In the presence of light, TiO_2_/Zn nanoparticles showed a better ability to control the bacterial leaf spot disease	[99]
32	TiO_2_	Imidacloprid	Nanocapsules	Increased photochemical stability and prolonged release of encapsulated imidacloprid	[100]
33	Silver	AgNPs	Nanoparticles	The nano-size silver colloidal solution has antifungal properties which inhibit the fungal growth	[101]
34	Copper	CuNPs	Nanoparticles	Copper nanoparticles are an effective and environment friendly strategy to control bacterial blight of pomegranate	[102]
35	Alumina	Nano-structured alumina	Nanoparticles	A safe, reliable, and cheap nano-structured material to control insect pests	[103]
36	CaCO_3_	Validamycin	Nanoparticles	Sustained release with good germicidal efficacy and environmentally compatible	[104]
***Layered double hydroxide (LDH) based:***					
37	Zinc-aluminium-LDH (ZAL)	3, 4-dichlorophenoxyacetic acid	Nanocomposite	Successfully intercalated into the ZAL layer; a potential carrier for CRF 3, 4-dichlorophenoxyacetic acid in environmentally friendly agrochemicals	[105]
38	Zn–Al LDH (ZAL)	2,4-D	Nanocomposite	More sustained release and this characteristic depended on the type of anions and their concentrations in the release medium	(bin [106])
39	Organo/LDH	Alachlor and metolachlor	Nanocomposite	Organo/LDHs may act as suitable supports for pesticide slow-release formulations with the aim of reducing adverse effects on soil and the environment	[107]
40	Mg–Al hydrotalcite (HT)	Terbuthylazine	Nanocomposite	Retards the release of terbuthylazine into aqueous solution and reduced herbicide leaching	[108]
41	Mg/Al–NO_3_ LDHs	2,4-D	Nanocomposite	Adsorption characteristics of 2,4-D are significantly different in different layer charge densities	[109]
42	Calcined hydrotalcite (HT)	2,4-D, clopyralid and picloram	Nanocomposite	The intensity of the adsorption is related to the acidity of the pesticides	[110]
43	Calcined LDHs	2,4-D	Nanocomposite	Calcined LDH possessed a very high capacity to adsorb 2,4-D	[111]
44	Mg/Al layered double hydroxides	Atrazine	Nanohybrid	The herbicide-containing nanohybrid could enable slow, controlled release of the herbicide	[112]
45	Mg/Al LDH	2,4-D, MCPA and picloram	Nanocomposite	CRF of 2,4-D, MCPA, and picloram within Mg/Al LDH minimise rapid herbicide leaching losses	[113]
46	Cu–Fe-LDH	2,4-D	Nanocomposite	Good adsorbent of 2,4-D and maximum adsorption takes place at pH 4	[114]
47	LDHs	Imazamox	Nanocomposite	A good host material to obtain imazamox nano-formulations for use in controlled release, smart delivery systems to minimise leaching losses and hence water contamination	[115]
48	Zn–Al LDH	2,4-D and 4-chlorophenoxy acetate	Nanocomposite	Acts as a host matrix for the intercalation of guest anions to synthesise hybrid organic inorganic nanocomposites	[116]
49	LDHs	MCPA	Nanocomposite	Excellent adsorbent and MCPA complexes based on LDHs support controlled release, which reduces herbicide leaching in soil	[117]
50	Mg/Al layered double hydroxides	MCPA	Nanocomposite	Good adsorbent of MCPA and the adsorption capacity increases as the layer charge density changes	[118]
51	Zn layered hydroxide (ZLH)	2,4-D	Nanohybrid	Good host material for 2,4-D intercalation and increases thermal stability	[119]
52	Zn–Al LDH (ZAL)	4-(2,4-dichlorophenoxy) butyrate and 2-(3-chlorophenoxy) propionate	Nanohybrid	Good host material for intercalation of phenoxy-herbicides with initial rapid release which is slower under equilibrium conditions	[120]
53	Zn–Al LDH (ZAL)	2,4-D and glyphosate	Nanohybrid	A suitable intercalating material with slow-releasing properties which reduce leaching of herbicide	[121]
54	Zn–Al LDH (ZAL)	Dichlorprop	Nanohybrid	Enhanced adsorption capacity with slow-release properties	[122]
55	Mg/Al LDH	MCPA	Nanohybrid	Enhanced adsorption capacity with slow-release properties	[123]
***Clay-based:***					
56	Montmorillonite and wheat gluten	Ethofumesate	Nanocomposite	Higher sorption capacity and slow-release characteristics. Wheat gluten formulations (with or without montmorillonite) were more effective than commercial formulations	[124]
57	Smectite modified with biopolymer chitosan	Imazamox	Nanocomposite	Increases the adsorption of herbicide Imazamox at low pH and lowers the release rate of herbicide in water or soil-water suspension compared to commercial formulations	[125]
58	Montmorillonite-chitosan	Clopyralid	Bio-nanocomposites	Good herbicide adsorption capacity when the chitosan was in cationic form and the herbicide was in anionic form	[126]
59	Alginate-bentonite	Diuron	Nanogranules	Adding bentonite to the alginate-based formulation produced higher T50 values, indicating slower release of the diuron	[127]
60	Alginate-bentonite and anthracite	Chloridazon and metribuzin	Nanogranules	Alginate-based clays have higher sorption capacity and slow the release rate	[128]
61	Zeolite and bentonite	2,4-D	Nanocomposite	Compared with unmodified substrates, NCP-modified zeolite and bentonite showed higher sorption behaviour and slow-release formulations	[65]
62	Montmorillonite	Ethofumesate	Nanocomposite	Slow-release capacity and prevents photodegradation	[129]
63	Montmorillonite	Hexazinone	Nanocomposite	Good carrier materials for CRF of hexazinone herbicide, and reduce groundwater contamination	[45]
64	Halloysite tubes	Atrazine	Nanocomposite	Slow-release capacity and reduced leaching loss	[130]
65	Montmorillonite	Sulfentrazone and Metolachlor	Nanocomposite	Good herbicide loading-releasing behaviour, better weed control efficacy, reduced leaching	[131]
66	Montmorillonite	Bentazone and dicamba	Nanocomposite	Slow-releasing properties with similar herbicide efficacy to commercial formulations	[132]
67	Smectite	Bentazone	Nanocomposite	Good sorbent, controls leaching and runoff of herbicide with excellent weed control capacity	[133]
68.	Montmorillonite	2,4-D	Nanocomposite	Higher adsorption capacity with slow-release properties	[134]
69	Montmorillonite and carbonated hydrotalcite	Imazaquin	Nanocomposite	Adsorption percentages were very high (%Ads >95%) and high stability, reduced leaching and prolonged herbicide activity compared to others	[55]
70	Montmorillonite	Ethofumesate	Nanocomposite	Lower polarity enhances adsorption capacity, slow-release properties	[135]
71	Montmorillonite	2,4-D	Nanocomposite	Reduces the immediate release of active ingredients into groundwater and a good carrier material for slow-release formulation, minimising leaching loss	[136]
72	Sepiolite	Metribuzin	Nanocomposite	Retards herbicide mobility, slow-release properties along with higher weed control efficacy	[56]
73	Montmorillonite	Bentazone and dicamba	Nanocomposite	Slow-release formulation of anionic herbicides	[137]
74	Montmorillonite	Norflurazon	Nanocomposite	Higher adsorption capacity, enhances photo-stabilisation, higher weed control efficiency compared to commercial formulations	[138]
75	Montmorillonite	Norflurazon	Nanocomposite	Adsorption is directly proportional to herbicide concentration and release, with a smaller concentration lasting longer	[139]
76	Montmorillonite	Picloram	Nanocomposite	Higher adsorption and slow-releasing properties	[140]
77	Montmorillonite	Acetochlor	Nanocomposite	Improved herbicide activity	[141]
78	Montmorillonite	Simazine	Nanocomposite	Higher adsorption capacity, slow-release properties, reduced leaching loss and similar herbicide efficacy to commercial formulations	[142]
79	Montmorillonite	Picloram	Nanocomposite	Retards the vertical movement of herbicide and reduces leaching	[143]
80	Kaolinite	Amitrole	Nanocomposite	High loading capacity and slow-release properties	[144]
81	Smectite	Imazamox	Nanocomposite	Increases adsorption at low pH and slows the release rate of herbicide in water	[125]
82	Halloysite and kaolinite	Amitrole	Nanocomposite	Enhances adsorption and slow-release properties	[145]
83	Montmorillonite	Hexazinone	Nanocomposite	Slow-release properties, excellent herbicidal efficacy, and reduced leaching	[67]
84	Montmorillonite	Imazaquin	Nanocomposite	Higher adsorption capacity, slow-release capacity	[146]
85	Zeolite and bentonite	2,4-D	Nanocomposite	High adsorption capacity, slow-release capacity, environmentally friendly approach to weed control	[26]
86	Montmorillonite and clinoptilolite	Paraquat	Nanobeads	Good host material for CRF of paraquat with long-term herbicidal activity	[147]
87	Montmorillonites and hydrotalcite	Imazamox	Nanocomposite	Higher sorption rate and slow-release capacity	[148]
88	Montmorillonite	Alachlor, atrazine and trifluralin	Nanocomposite	Improved CRF, reduced leaching loss	[149]
89	Bentonite and montmorillonite	Metolachlor	Nanocomposite	High adsorption capacity, reduced mobility of herbicides (upper 0–17 cm) and similar herbicide efficacy to commercial formulations	[150]
90	Smectite	Atrazine	Nanocomposite	Slow-release properties, reduced leaching loss and similar herbicide efficacy to commercial formulations	[151]
91	Montmorillonite	Ethofumesate	Nanocomposite	Slow-release of herbicide compared to commercial formulations	[124]
92.	Montmorillonite	2,4-D	Nanocomposite	Enhances adsorption with slow-release properties	[152]
93	Montmorillonite	Diuron	Nanocomposite	Effective adsorbent for organic compounds in water	[153]
94	Montmorillonite	Acetochlor	Nanocomposite	Higher adsorption capacity, slow-release properties and good weed control efficacy	[154]
95	Bentonite	Metolachlor	Nanocomposite	Excellent adsorption capacity and slow-release properties	[155]
96	Montmorillonite and sepiolite	Trifluralin and norflurazon	Nanocomposite	Increased photostabilisation and reduced leaching loss	[156]
97	Montmorillonite	Fluridone	Nanocomposite	Better adsorption and slow-release formulation	[157]
98	Montmorillonite	Alachlor and metolachlor	Nanocomposite	Prevents volatilisation and photodegradation, prolongs herbicidal activity	[158]
99	Smectite	Dicamba	Nanocomposite	Sorption is favoured for high-layer charge and saturation with organic cations close to CEC	[47]
100	Smectite	Fenuron	Nanocomposite	Slow-release with similar efficacy to commercial formulations, reduces runoff and leaching loss	[159]
101	Sepiolite	Alachlor or Metolachlor	Nanocomposite	Enhances adsorption and reduces volatilisation	[160]

Our knowledge of the combined toxicity of nano-delivered pesticides and carrier materials on non-target living organisms remains poor. Toxicological data on nanomaterials is scant, particularly regarding their chronic effects on human health [[161], [162], [163]]. In future, serious health and safety issues may arise from these new pesticides. To overcome these uncertain problems, clays could be considered as a novel carrier material for herbicide CRF due to their multidimensional properties, including easy availability, low cost, large surface area, good intercalation chemistry, high surface reactivity, lack of toxicity, as well as their biodegradable, ubiquitous, natural occurrence and eco-friendly characteristics [52,164,165]. It is important to note that the preparation of organically modified clay, including nano-clay and clay-based nanocomposites, has enhanced the preparation of CRF due to the alteration of clay surfaces from hydrophilic to hydrophobic.

**Fig. 10:**
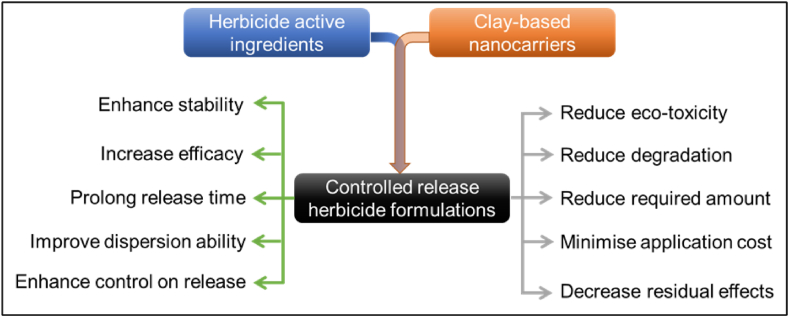
Expected outcome of clay-based CRF.

Over the last two decades, the preparation and characterisation techniques for organoclays have progressed well; however, their utilisation in CRF preparation is still in its early stages. For the preparation of clay-based CRF, it is important to ensure sufficient loading of active ingredients onto the clay moieties as well as their controlled release. Generally, active ingredients are loaded onto an organoclay via the sorption process, establishing various interactions including cation exchange, ligand exchange, hydrophobic interactions, hydrogen bonding, protonation, and water bridging. The interactions between clays and active ingredients also influence the release behaviour of the active ingredients. It is crucial to design specific interaction site(s) tailored organo- and/or nano-clays as well as nanocomposites. Further investigations are required to reveal the wide-ranging benefits of this approach in the next few decades, if not sooner. Some expected improved features of clay-based nano-herbicide formulations are presented in Fig. 10.

## 3 Scope for future research

Considering the ecotoxicological aspect, it is crucial to formulate eco-friendly herbicide formulations, which will have less adverse effects to the environment and biota. Utilising the benefits of nanotechnology, a safer formulation can be synthesised through CRF. The application of nanotechnology in CRF of herbicide is in the early stages, and there are still a number of areas for future research to synthesise eco-friendly CRF of herbicide. Currently, various carrier materials including clays, different polymers, metals and their oxides, are used to synthesise CRF of various herbicides. Different surfactants-modified organoclays have been used as carrier materials, whereas there is limited information on application of zwitterionic surfactants to modify pristine clays. Zwitterionic surfactants are deemed to be less toxic than anionic and/or cationic surfactants, hence research should be pursued to modify pristine clays and assess their suitability as carriers for CRF of different pesticides. There is now a much wider scope to synthesise eco-friendly natural carrier materials for CRF of herbicides that will have zero toxicological effects on the environment and living organisms. Synthesis of new and eco-friendly nanocarriers for CRF of various pesticides (herbicide, insecticides, fungicides etc.) and fertilisers, along with their pest control efficacy and combined eco-toxicity, have a wide scope for future research.

## 4 Nanotechnology in food and agriculture: impact and future awareness

Nanotechnology deals with minute things sized from 1 to 100 nm, but it is now an indispensable part of our daily lives due to its wide application [52]. The worldwide implementation of nanotechnology is expanding in almost every industry or sector, including chemistry, physics, energy, electronics, textiles, cosmetics, pharmaceutical science, medicine, material science, and food and agriculture, which is changing people's attitudes toward it in a positive way [24,53]. However, the application of nanotechnology in the food industry and agriculture began in earnest on September 9, 2003, with the inauguration of the first roadmap for this strategy by the United States Department of Agriculture [52,166].

A project was conducted by the United States Department of Agriculture in 2003, where the main focus was to assess nanotechnology for its efficient use in pesticides (herbicides, insecticides, fungicides etc.), fertilisers and plant growth regulators, to produce more food with equivalent quality, nutritional value, and safety. This approach is known as “precision farming”, where yield and quality will be improved but without environmental damage to soil or water, etc. [24,52]. Currently, nanotechnology is part of every agricultural/horticultural practice, such as production, processing, packaging, storage, transportation, and even delivery to our plates. Various nanomaterials have been devised for improving crop growth, production, quality, and safety, as well as for monitoring environmental conditions [52]. However, while nanotechnology is now making enormous progress in the scientific world, we know little about its toxicological impacts on the environment and biodiversity.

Certain detrimental substances from nano-pesticides may enter the human body through edible plant parts and animals via the food chain. Therefore, the risks to human health and for chronic diseases are evident [161], and there are also diverse toxicological effects on plants, animals, and the environment to consider [57,167]. Nano-pesticides are nonetheless a new and important formulation given their superior efficacy compared to conventional formulations [24]. So far, in the nano-pesticide delivery system, most of the emulsifiable concentrates are formulated using polar solvents including alcohols, ketones, and benzenes, which are acutely toxic and can easily move into the soil and mix with groundwater. Degradation of these polar solvents is currently very difficult, if not impossible to achieve, which can result in acute poisoning and even death [161].

The direct use of nanotechnology, despite its use in human food, is restricted except for iron oxide and titanium oxide, which are used as a colourant and a food pigment, respectively [52]. Food manufacturers may hide the chemical information of their new products and keep their technology secret for reasons such as business privacy, market share, competition, etc. [168]. Some food manufacturing companies are reluctant to disclose the existence of nanomaterials in their products. Several nanomaterials are used as food additives and functional or nutritional ingredients. Currently, silver nanoparticles are widely used in the packaging of milk and milk products, and people are consuming these without knowing, which is unacceptable in both the legislative and ethical sense. Direct exposure to these nanomaterials may pose a threat to human health [52,168,169]. Additionally, nanomaterials may accumulate at higher concentrations in the human body and edible parts of plants, thereby increasing the risk of disease or other serious medical problems [170,171]. To evaluate the toxicological effects evident in France, titanium dioxide (TiO_2_) nanoparticles were fed to rats for more than 100 days and carcinogenic effects were detected. Due to the noxious effects of TiO_2_, the French Food Safety Agency decided to ban the import and marketing of TiO_2_ by 2020 [52].

A case study in Singapore reported that people have negative perceptions of nanotechnology, and awareness is growing about the potential side-effects [172]. It is not surprising that most agrifood manufacturers have little or no knowledge of nanotechnology and even less awareness of the potential adverse health and environmental effects [173]. During manufacture, agrifood organisations should evaluate the toxicological effects of nanomaterials on human cells, tissues, and organs, as well as the migration and accumulation of nanomaterials in food and the food chain, their degradation, adsorption and bioaccumulation channels, and what happens when they enter the natural environment. The European Commission and United States Food and Drug Administration are the world's leading authorities responsible for the regulation and legislation of food nanotechnology and should monitor food-producing organisations regularly [52].

## 5 Conclusion

Weed management is an important and continuous process throughout crop cultivation to ensure quality and higher production. Of the various weed control techniques, herbicidal weed management is more effective and reliable to the farmers. Improvements in current herbicide formulations is highly desirable, where CRF are alternative and effective way to compensate the toxicological effects associated with available commercial herbicides formulations. This manuscript focused on the history, progress, and consequences of herbicide application, and discussed potential ways to reduce eco-toxicity due to herbicide application. Selection of suitable carrier materials stands first to synthesis effective CRF of herbicide. This review directed the ways to use eco-friendly carrier materials for CRF to reduce eco-toxicity due to chemical herbicides. So far, few different carrier materials have been used in CRF of herbicide, whereas utilisation of eco-friendly carrier materials in synthesising CRF of herbicide is important to protect our environment. Future research should be focused on synthesising eco-friendly natural carrier materials for CRF of herbicides that will have zero toxicological effects on the environment and living organisms. Moreover, research should be focused on investigation of long-term toxicological effects of synthesised new formulations on soil flora and fauna.

## CRediT authorship contribution statement

**Santosh Kumar Paul:** Writing – review & editing, Writing – original draft, Conceptualization. **Santa Mazumder:** Writing – review & editing. **Ravi Naidu:** Writing – review & editing, Writing – original draft, Supervision.

## Declaration of competing interest

The authors declare that they have no known competing financial interests or personal relationships that could have appeared to influence the work reported in this paper.
